# Efficacy and safety of chloroquine plus primaquine for the treatment of *Plasmodium vivax* malaria in Hamusit site, Northwestern Ethiopia

**DOI:** 10.1186/s12936-024-05031-9

**Published:** 2024-07-06

**Authors:** Habtamu Gebrie, Mulat Yimer, Animen Ayehu, Hussien Mohammed, Henok Hailgiorgis, Yonas Wuletaw, Mesay Hailu, Getachew Tolera, Geremew Tasew, Mogess Kassa, Bokretsion Gidey

**Affiliations:** 1 Department of Medical Laboratory Science College of Medicine and Health Science, Dilla University, Dilla, Ethiopia; 2 Department of Medical Laboratory Science College of Medicine and Health Science, Bahir Dar University, Bahir Dar, Ethiopia; 3 Ethiopian Public Health Institute, Addis Ababa, Ethiopia

**Keywords:** Chloroquine, Drug efficacy, Ethiopia, *Plasmodium vivax*, Primaquine

## Abstract

**Background:**

*Plasmodium vivax* malaria is still an important public health problem in Ethiopia. Unlike *Plasmodium falciparum, P. vivax* has a dormant liver stage (hypnozoite) that can be a risk of recurrent vivax malaria unless treated by radical cure with primaquine. Drug resistance to chloroquine is threatening malaria control and elimination efforts. This study assessed the therapeutic efficacy and safety of chloroquine plus 14 days of primaquine on *P. vivax* infection based on parasitological, clinical, and haematological parameters.

**Methods:**

A single-arm in vivo prospective therapeutic efficacy study was conducted to assess the clinical and parasitological response to the first-line treatment of *P. vivax* in Ethiopia, chloroquine plus 14 days low dose of (0.25 mg/kg/day) primaquine between December 2022 and March 2023 at Hamusit Health Centre using the standard World Health Organization (WHO) protocol. A total of 100 study participants with *P. vivax* mono-infection who were over 6 months old were enrolled and monitored for adequate clinical and parasitological responses for 42 days. The WHO double-entry Excel sheet and SPSS v.25 software were used for Kaplan–Meier survival analysis, and a paired t-test was used for analysis of haemoglobin improvements between follow up days.

**Results:**

A total of 100 patients were enrolled among those, 96% cases were rural residents, 93% had previous malaria exposure, and predominant age group was 5–15 years (61%). 92.6% (95% CI 85.1–96.4%) of enrolled patients were adequate clinical and parasitological response, and 7.4% (95% CI 3.6–14.9%) recurrences were observed among treated patients. The fever and parasite clearance rate on day 3 were 98% and 94%, respectively. The baseline haemoglobin levels improved significantly compared to those days 14 and 42 (p < 0.001). No serious adverse event was observed during the study period.

**Conclusions:**

In this study, co-administration of chloroquine with primaquine was efficacious and well-tolerated with fast resolution of fever and high parasites clearance rate. However, the 7.4% failure is reported is alarming that warrant further monitoring of the therapeutic efficacy study of *P. vivax*.

## Background

Malaria is a vector-borne protozoal disease caused by different *Plasmodium* species, of which *Plasmodium falciparum* and *Plasmodium vivax* are the predominant ones [1]. Eighty-five countries are malaria-endemic; 3.4 billion people are at risk of malaria; 249 million cases and 608,000 deaths were reported globally; and in Africa, 233 million and 580,000 cases and deaths were also reported by the World Health Organization (WHO) [2]. In Ethiopia, around 68% of the population lives in malaria-risk areas, and more than 1.2 million cases were reported in 2022 [3, 4]. Ethiopia has made significant gains in malaria control, which led to the development of a guideline to eliminate malaria from low transmission areas and reduce the burden in high and moderate transmission settings [5].

Anti-malarial drug resistance is a threat to effective malaria control and elimination around the world. Several studies indicated that chloroquine (CQ) resistance spread across most *P. vivax-*endemi*c* regions [6]. The management of *P. vivax* cases has been challenging due to the hidden and recurring liver stage (hypnozoite) [7]. The national malaria treatment guideline in Ethiopia recommends the use of 0.25 mg/kg/day of a 14-day low dose of primaquine for radical cure with close clinical and laboratory follow up at health facility. Recently, various findings in Ethiopia stated that the recurrence of *P. vivax* infection after treatment is above 10% (failure rate of CQ plus PQ was 10.9 (95% CI 5.8–19.9%) [8] and 13% [95% CI 7.4–21.7%] [9]. This failure rate is beyond the cut-off limit that was recommended by the WHO in 2009.

In Ethiopia, *P. falciparum* resistance to chloroquine was first noted in the 1970s and was followed by resistance to sulfadoxine-pyrimethamine [10], yet, chloroquine remains effective and is used as the first-line anti-malarial treatment for *P. vivax* infection. The WHO recommends the regular monitoring of the efficacy of anti-malarial drugs [11]. This study aimed to determine the cure rate of chloroquine plus 14 days of primaquine on patients with *P. vivax* malaria over 42 days of follow-up periods in the study setting.

## Methods

### Study design and area

**Fig. 1:**
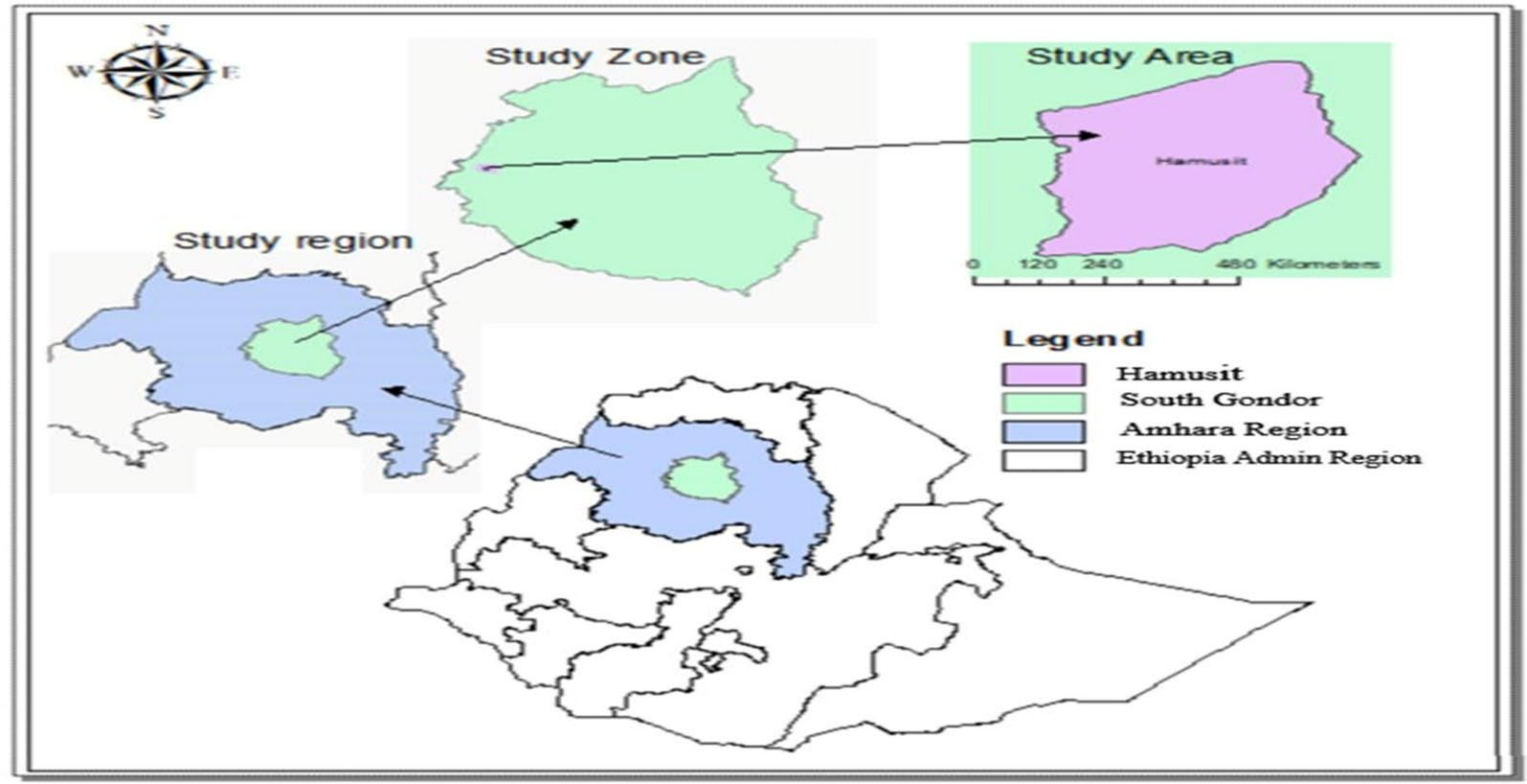
Map of study area

A single-arm in vivo prospective therapeutic efficacy study was conducted at Hamusit Health Centre, South Gondar Zone, Northwestern Ethiopia, from December 2022 to March 2023. The climate of the study area is temperate, and the altitude is 2077 m above sea level. Hamusit has 1300 mm of mean annual rainfall and 26 °C mean annual temperature. The study area (Fig. 1) has intensive irrigation for the cultivation of rice, onions, and sugarcane. The Gumara River crosses the study area and Lake Tana, the biggest lake in Ethiopia, borders the catchments of the study area. Malaria transmission occurs throughout the year, and the predominant *Plasmodium* species are *P. falciparum* and *P. vivax*. Approximately 55,426 catchment populations receive health services at Hamusite Health Centre [12].

### Study population, inclusion and exclusion criteria

Outpatients aged more than 6 months who visited the Hamusite Health Centre with suspected uncomplicated malaria (axillary temperature ≥ 37.5 °C or history of fever during the previous 48 h) were tested for malaria parasites and recruited if they had *P. vivax* mono-infection with a parasite density of ≥ 250 asexual parasites/μL by microscopic examination. Study participants must live within a 10 km radius of the health centre; and consented and/or assented to participate in the study. Patients were not enrolled if they took drugs that may interfere with malaria treatment and had the intention to move away from the study catchment during the follow-up period of 42 days; evidence of severe malaria, known hypersensitivity to the study drugs, previous history of haemolysis, and pregnant or women breastfeeding a child ≤ 6 months old.

### Sample size

The sample size was determined according to the WHO [11] guideline for anti-malarial drug efficacy study. Assuming a treatment failure of 5% with a 95% confidence level and 5% precision, a minimum sample of 73 patients was estimated. With 20% added to allow loss to follow-up and withdrawals during the 42 days of follow-up periods, 88 patients were targeted.

### Laboratory procedures

Thick and thin blood smears were taken from all participants and prepared on the same slide for detection of parasites at all-time points (on day 0, 1, 2, 3, 7, 14, 21, 28, 35, 42) during the follow-up period. Smears were stained with 10% Giemsa for 10 min and examined with a light microscope by two well-trained laboratory technicians. Parasite densities were calculated from thick blood smears by counting the number of asexual parasites against the 200 WBCs. Parasitaemia was estimated by assuming a WBC count of 8000/µl. Gametocytes were detected and counted on a thick film [11].

Haemoglobin concentrations were measured using a portable spectrophotometer (HemoCue®, Anglom, Sweden) on days 0, 14, 28, and 42. After measuring the haemoglobin level, anaemia is defined according to the WHO classification: Hgb > 11 g/dl for < 5 years, > 11.5 g/dl for 5–15 years, and > 13 g/dl for adult males indicate normal. Hgb ≤ 5.0 g/dl was considered severe anaemia and an exclusion criterion [11, 13].

### Treatment and follow-up

Eligible patients were given a standard therapeutic oral CQ and PQ radical treatment according to the national malaria treatment guideline of Ethiopia [9]. Total of 25 mg base per kg of CQ over 3 days (10 mg base/kg on Days 1 and 2, and 5 mg base/kg on Day 3) of 500 mg CQ tablets (Batch number: L8080013, manufacturer was Ipca Laboratories Ltd) and 7.5 mg base per kg of PQ tablet (Batch number 92147, manufacturer Remedica Ltd) medication given as 0.25 mg/kg daily for 14 days. All CQ drugs administered in the facility were under supervision by the study staff. But for PQ, patients were observed for the first 3 days and the remaining doses were taken at home, indirectly observed by questioning. Participants were followed over 42 days (i.e. Day 0, 1, 2, 3, 7, 14, 21, 28, 35, 48) (Table 1).

**Table 1:** Follow up days

S. no	Procedures	D 0	D 1	D 2	D 3	D 7	D 14	D 21	D 28	D 35	D 42	Extra day
	Adverse event	X	X	X	X	X	X	X	X	X	X	
1	Temperature	X	X	X	X	X	X	X	X	X	X	
2	BF(asexual parasites count)	X	X	X	X	X	X	X	X	X	X	
3	Gametocyte count	X	X	X	X	X	X	X	X	X	X	
4	Hemoglobin	X					X		X		X	
5	DBS	X			X	TF	TF	TF	TF	TF	TF	
6	CQ treatment	X	X	X								
7	PQ treatment	X	X	X	X(3)	X(6)						
8	Urine/hill men	X	X	X	X	X	X					
9	Treatment outcome											

Patients were observed for 60 min after treatment for vomiting. Patients who vomited their medication within the first 30 min received a repeat full dose, while those vomiting within 30–60 min received a half dose. Patients who vomited twice were referred to a higher level of care for management with parenteral artesunate therapy and withdrawn from the study. All study drugs were obtained from the Ministry of Health. Noninterfering concomitant treatments were given to control fever (> 38 °C) and other ailments, as required [11, 12].

### Classification of treatment outcomes

The primary outcomes were treatment failures and adequate clinical parasitological response (ACPR) on day 42 treatment outcome of the participants per WHO protocol [11], treatment responses were classified as early treatment failure (ETF), late clinical failure (LCF), late parasitological failure (LPF) or adequate clinical and parasitological response (ACPR). Secondary outcomes are any clinical and parasitological outcomes following chloroquine plus primaquine treatment (parasite, fever, gametocyte clearance rate, incidence of drug adverse events).

### Clinical evaluation

A standard physical examination was performed at baseline (day 0 before dosing) and on days 1, 2, 3, 7, 14, 21, 28, 35, and 42 [11]. All patients were asked routinely about previous symptoms and about symptoms that have emerged since the previous follow-up visit, and Hillmen urine tests were done up to day 14 (on days 1, 2, 3, 7, 14) to check for any haemolysis. When clinically indicated, patients were evaluated and treated appropriately.

### Data quality management and analysis

The study protocol was adhered and all data were collected and recorded correctly on the case record form. Laboratory and clinical data were recorded daily on the case record form designed for the study. Any change or correction to a case record form was dated and explained. All case record forms were checked for completeness. All data from enrolled patients was imported into the WHO Excel sheet (double entry) and analyzed to determine the therapeutic efficacy of CQ plus PQ. Data was also entered into SPSS (version 25) software to calculate descriptive statistics (mean, standard deviations, and range).

A paired sample t-test was used to compare the mean Hgb level between D0 and D14, D0 and D28, D0 and D42, D14 and D28, D14 and D42, and D28 and D42. All comparisons were performed at a 95% CI and a significance level of 0.05. Kaplan–Meier (K–M) survival analysis and per-protocol analysis were used for the estimation of primary outcomes and secondary outcomes. Five percent of the slides were read by WHO-qualified microscopists for quality control, and the reliability of the scales (balance, thermometer, HemoCue, and HCG strips) was verified before the study began and checked at regular intervals.

### Ethical consideration

The study ethical clearance was obtained from the Ethical Review Board of the College of Medicine and Health Sciences at Bahir Dar University (IRB) and from institutional review board of the Ethiopian Public Health Institute (EPHI) before its initiation. In addition, permission letters were obtained from the Amhara Public Health Institute (APHI) and Hanusit Health Centre. Written informed consent was obtained from adult patients, while for children, informed assent was secured from their parents or guardians. The confidentiality of the results was maintained, and patients had the right to stop or withdraw from the study at any time [11].

## Results

### Baseline characteristics

**Fig. 2:**
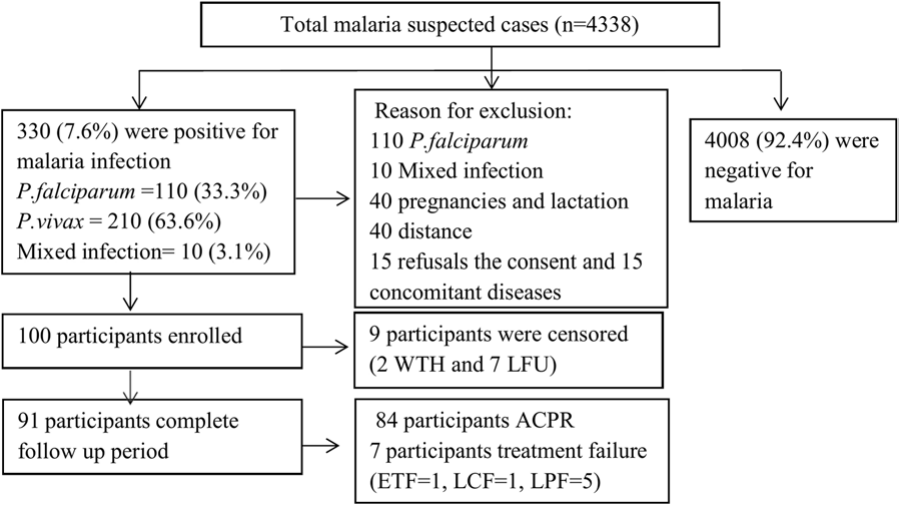
Flow chart of participants recruitment for 42 days of follow-up. Among 210 P. vivax mono infections: 40 pregnancies and lactation, 40 far from the catchment area, 15 refused consent, and 15 had concomitant disease and 100 participants were enrolled. Nine participants were lost to follow-up and withdraw from the study and excluded from the Kaplan Meier analysis. Of 91 study participants seven were categorized under treatment failure. LFU, lost to follow up; WTH, withdrawal; ETF, early treatment failure; LCF, late clinical failure; LPF late parasitological failure; ACPR, adequate clinical and parasitological response

A total of 8263 patients attended the Outpatient Department (OPD) of Hamusit Health Centre during the study period. Of these, 4338 (52.50%) were suspected malaria cases. From these, 210 (4.84%) individuals were *P. vivax* confirmed cases. From *P. vivax-*positive individuals, 100 (47.62%) of the *P. vivax* mono-infection cases that fulfilled the inclusion criteria were recruited. From the total 100 enrolled participants, 91/100 (91%) completed the follow-up, and 9 participants were censored (2 (2%) withdrawn (WTH) and 7 (7%) lost from the follow-up (Fig. 2).

Among the total recruited participants, 96% cases were rural residents. Ninety-three had previous malaria history and used antimalarial drugs. The predominant age group was 5–15 years (61%). All study participants had a bed net. There was no hypersensitivity reaction during and after anti-malarial drug (CQ-PQ) treatment (Table 2).

**Table 2:** Demographic and clinical characteristics of study participants at the baselines of study

Characteristic	Value
Gender	
Female, n (%)	38 (38%)
Males, n (%)	62 (62%)
Residence	
Rural	96%
Urban	4%
Mean age years (range)	12 (0.8–60)
Weight(kg), mean(SD) (range)	25.4 (15.3) (6–70)
Age group, n (%)	
< 5 years n (%)	18 (18%)
5–15 years n (%)	61 (61%)
≥ 15 (adults) n (%)	21 (21%)
Axillary temperature (°C), mean(SD)	38.1 ± 1
Mean (range) asexual parasitaemia (per µL)	4423 (259–14,520)
Mean gametocytaemia, n (%)	26 (26%)
Mean Hb in g/dL, mean (SD) (range)	11.8 (1.97) (8.5–18.5)
Presence of bed net n (%) and bed net utilization n (%) with participants	100 (100%) and 100 (100%)
Previous malaria attach n (%)	93 (93%)
Antimalarial drug utilization n (%)	
AL	81 (81%)
CQ	12 (12%)

Baseline clinical characteristics

### Baseline clinical characteristics

**Fig. 3:**
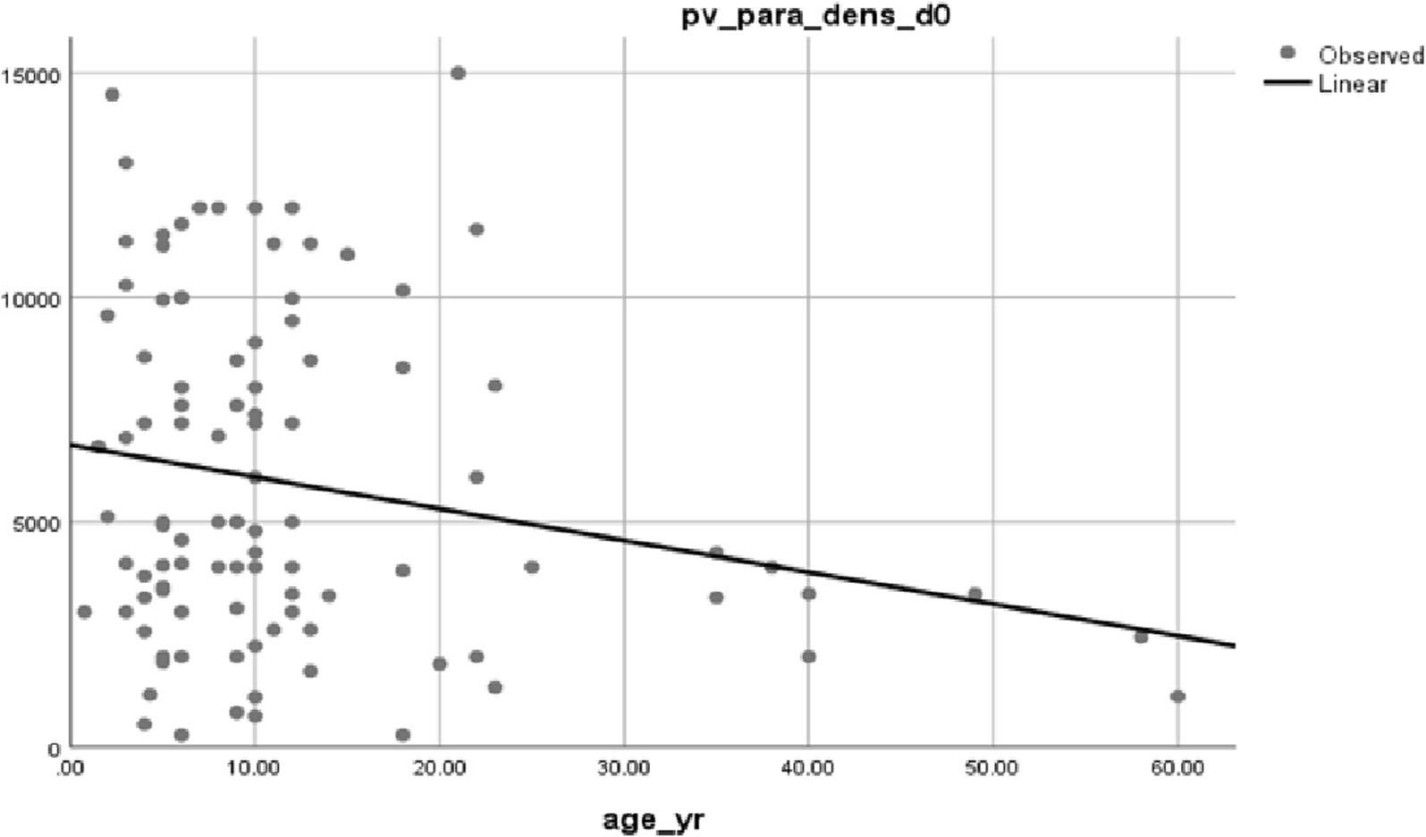
Relation between age and parasite density at baseline of study participants

At the baseline of the study, the mean asexual parasite density was 4423, with a range of 259–14,520, and about 26% of cases had gametocytes. The mean haemoglobin level (SD) was 11.8 g/dl ± 1.97 ranges (8.5–18.5 g/dl); in addition, 56% of the study participants were anaemic (Table 3). At the baseline of the study, parasite density and age of the participants were inversely proportional. Parasite load was higher in children than adults (P = 0.031) (Fig. 3**)**.

**Table 3:** Baseline anaemic status, mean parasite density and gametocyte carriage of participants

Variable	Sex		Residence		Age category			Total
	Female	Male	Rural	Urban	Under 5	5–15	Adult	
No (%)	38 (38%)	62 (6%)	96 (96%)	4 (4%)	18 (18%)	61 (61%)	21 (21%)	100 (100%)
Anemia status (%)								
Mild	12	14	25	1	7	11	8	26
Moderate	9	21	29	1	3	25	2	30
Total	21	35	54	2	10	36	10	56
	6261.1	5702	5904.4	4760	6368.3	5863.5	5117	4423
Mean (Geo) asexual parasite(µl)								
Gametocyte carriage n (%)	11	15	25	1	5	17	4	26

### Primary outcomes

#### Cure rate of chloroquine plus primaquine treatment

Based on the Kaplan–Meier analysis, the cure rate and failure rate of chloroquine (CQ) with primaquine (PQ) on 28 days of follow-up periods was 96% (95% CI 89.7–98.5%) and 4% (95% CI 1.5–10.3%), respectively. However, on the 42-days of follow-up periods, the cure rate of CQ –PQ treatment was 92.6% (95% CI 85.1–96.4%), with a failure rate of 7.4% (95% CI 3.6–14.9%) (Table 4).

**Table 4:** Chloroquine plus primaquine treatment outcome of the study participants based on K–M analysis

Follow up days	At risk	Censored	Failure	Survived	K–M survival rate	K–M failure rate
Day 0	100	0	0	100	1.00	0.00
Day 1	100	0	0	100	1.00	0.00
Day 2	100	1	0	99	0.99	0.00
Day 3	99	1	1	97	0.98	0.01
Day 7	98	2	0	95	0.97	0.00
Day 14	96	1	3	91	0.94	0.03
Day 21	95	1	0	90	0.92	0.00
Day 28	94	1	0	89	0.94	0.00
Day 35	93	2	1	86	0.92	0.01
Day 42	91	0	2	84	0.92	0.02
Cure and failure rates of the drug on 28 days	Total cure rate % (95% CI)			96% (95% CI 89.7–98.5%)		
	Total treatment failure % (95% CI)			4% (95% CI 1.5–10.3%)		
Cure and failure rates of the drug on 42 days	Total cure rate % (95% CI)			92.6% (95% CI 85.1–96.4%)		
	Total treatment failure % (95% CI)			7.4% (95% CI 3.6–14.9%)		

### Secondary outcome

#### Parasite clearance

Based on the per-protocol analysis, 94% of the study participants cleared parasitaemia on day 3 of the follow-up period. However, four cases developed parasitaemia before 28 days of follow-up periods (i.e. one case of ETF on day 3 and three cases of LPF on day 14), and three cases also developed parasitaemia after 28 days of follow-up (i.e. one case of LCF on day 35, and two cases of LPF on day 42). From the total of seven participants who developed treatment failure, five (71.4%) of the study participants had a higher parasitaemia than the baseline parasitaemia (Table 5).

**Table 5:** Parasitic density and its clearance of the participants treated with chloroquine plus primaquine

Types of treatment failure	Pt.ID	Sex	Age (yr)	FUD	Parasitic density				Ratio/µl of para. dens at day of treatment failure to day 0	
					At day 0 (baseline)/µl		At the day of treatment failure/µl			
ETF	506	M	6	Day 3	4080		5300		1.3	
LCF	546	M	6	Day 35	8501		8000		0.9	
LPF	517	M	4	Day 42	2560		580		0.2	
	518	M	10	Day 42	2240		8960		4.0	
	528	M	21	Day 14	2640		6000		2.3	
	559	M	11	Day 14	2600		3590		1.4	
	598	F	9	Day 14	780		800		1.03	
FUD	Day0	Day1	Day 2	Day 3	Day 7	Day 14	Day 21	Day28	Day 35	Day 42
Gam carrier (n)	26	9	0	0	0	0	0	1	1	0
Presence of asec para (n)	100	54	11	6	0	3	0	0	1	2

#### Fever clearance

Almost 98% of the study participants were cleared of their fever on day 3. The fever clearance rate had a significant association with the parasite clearance rate on day 3 (P = 0.033). However, some participants manifested a fever on different follow-up days (Table 6).

**Table 6:** Fever clearance of the study participants treated with chloroquine plus primaquine

Follow up days	Day 0	Day 1	Day 2	Day3	Day 7	Day 14	Day 21	Day 28	Day 35	Day 42
Mean body T^0^	38.1	36.6	36.0	36.0	36.0	36.1	36.1	36.1	36.2	36.1
No. Pts.T^0^ ≥ 37.5^0^c (n)	84	14	2	2	1	1	1	2	2	4

No.pts.T^0^ = Number of patients whose body temperature greater than 37.5 ^0^c (febrile)

The haemoglobin measurement of the study participants decreased following treatment. At the baseline of the study, 56 study participants were anaemic, but after following the treatment, their numbers decreased to 42, 21, and 16 on days 14, 28, and 42 of the follow-up periods, respectively. The mean haemoglobin level with standard deviation was improved following the treatment: 11.8 g/dl ± 1.97, 11.9 g/dl ± 1.4, 12.5 g/dl ± 1.3, and 12.7 g/dl ± 1.27 on days 0, 14, 28, and 42 follow-up days, respectively. There was significant improvement between days 0 and 28 (P = 0.01), days 0 and 42 (P < 0.0001), days 14 and 28 (P < 0.0001), and days 14 and 42 (P < 0.0001) (Table 7).

**Table 7:** Haemoglobin recovery

Follow up days	Mean hb (min–max) g/dl	P value
Day 0	11.7 (8.5–18.5)	< 0.001
Day 14	11.8 (7.9–15.3)	
Day 28	12.5 (9.5–16.5)	
Day 42	12.7 (8.6–16.9)	

^*^hgb haemoglobin, min minimum, max maximum, gram per decilitre

Of the total 56 anaemic cases, only 2 participants had not had a previous malaria attack at the baseline of the study, and all of them improved from their anaemia at the end of the follow-up period. At the baseline of the study, of the total 56 anaemic study participants, about 54 had a history of previous malaria attacks. Of these, 39 participants improved from anaemia at the end of the study (P = 0.513).

### Clinical signs, symptoms and adverse events after combination of chloroquine and primaquine treatment

The baseline mean ± SD body temperature was 38.1 ± 1 and the highest body temperature was recorded for under-five children (40.5 °C) and the minimum body temperature was recorded for the 5–15 age categories (35.5 °C). On day 3, about 98% of participants had cleared their fever. At the baseline of the study, 78% had a fever or reported a history of fever in the last 48 h. The remaining 22% of the participants had only a history of fever in the last 48 h and headache, joint pain, and abdominal pain were the most common clinical signs and symptoms, accounting for 94%, 40%, and 19%, respectively. After treatment, some adverse events were observed: mouth ulcer (1%), cough (1%), abdominal pain (2%), and nausea (1%) (Table 8).

**Table 8:** Clinical signs and symptoms, and adverse events after treatment with chloroquine plus primaquine

Adverse events and clinical sign and symptoms	Follow up days									
	Day 0	Day 1	Day 2	Day3	Day 7	Day 14	Day 21	Day 28	Day 35	Day 42
Fever (%)	100	5	1	2	1	3	2	0	2	1
Chills (%)	6	0	0	0	0	0	0	0	0	1
Headache (%)	94	3	2	5	2	4	2	2	1	1
Nausea n (%)	3	1	0	0	0	0	0	0	0	0
Vomiting (%)	8	0	0	0	0	0	0	0	0	0
Cough (%)	3	0	0	1	0	0	0	0	0	0
Joint pain (%)	40	0	0	1	0	0	0	0	0	0
Mouse ulcer (%)	0	0	0	0	1	1	0	0	0	0
Dark urine (%)	0	0	0	0	0	0	0	0	0	0

^*^Urine colour grade using Hillmen urine colour chart, all participants were recorded < 5

## Discussion

It is challenging to discern between recrudescence, reinfection, and relapse in *P. vivax* recurrent infections. Since the WHO reported in 2009 that over 50% of recurrence parasite genotypes varied from the parasite genotype at the baseline of the study (day 0), further research at Arbaminch, Ethiopia in 2023 revealed that 77.7% of recurrence parasite genotypes differed from the baseline (day 0) parasite genotype [8, 11]. For these reasons, the CQ-PQ treatment's cure rate in the current investigation was PCR uncorrected.

In the current study, the therapeutic efficacy of CQ-PQ on days 28 and 42 follow-up periods was 96% (95% CI 89.7–98.5%) and 92.6% (95% CI 85.1–96.4%), respectively. This finding indicates that CQ-PQ is highly efficacious. While, the discrepancy between the cure rates of CQ-PQ on day 28 and 42 might be, due to either reinfection being high on day 42 since, the removal of blood level concentration of CQ after 35 days may be higher, it might be also due to anti-malarial drug resistance or relapse [14]. The secondary outcomes also parasite clearance and fever clearance rates were 94% and 98% on day 3, respectively, and the haemoglobin level was significantly improved (p < 0.001) with no serious adverse events (Table 7).

The cure and failure rate of CQ-PQ on day 28 in the current finding were (96% (95% CI 89.7–98.5%)) and (4% (95% CI 1.5–10.3%)), respectively, which is in line with the two findings in Northeastern Myanmar 97.4% and (2.6%), and 94.8% and (5.2%), respectively [15, 16]. The cure rate and failure rate of this study on 42 days of follow-up period also (92.6% (95% CI 85.1–96.4%)) and (7.4% (95% CI 3.6–14.9%)), respectively, that is similar with the systematic review cure rate and failure rate findings, respectively in the world (i.e. 95.1% and 4.9%) and northeastern Myanmar (92.02% and 7.98%) [15, 17]. However, the therapeutic efficacy of CQ-PQ on 28 follow-up days in the current study was less than the findings in India (99.2%), Ethiopia (99.25%), and Colombia Pacific region (98.6%) [18–20]. Similarly, on the 42 days of follow-up periods efficacy of these anti-malarial drugs in the current study was also less than the findings in Kolkata, India (100%) [21].

Treatment with primaquine is crucial for preventing relapses because it eliminates the liver's hypnozoite and gametocyte stage. Primaquine failure is verified by blood schizonticide clearing blood-stage parasites; also, study subjects need to be closely monitored during therapy and shielded from reinfection. The medication's quality, participants' ages, treatment dosages, and patients' preexisting anti-malarial immunity all have an impact on the medication's effectiveness [12, 17, 22]. Since both primaquine and chloroquine were administered to study participants, it was assumed that primaquine eliminated the gametocytes and liver-stage parasites, while chloroquine cleaned the blood-stage parasites. The anti-malarial drugs were also obtained from the government and treated based on the national treatment guidelines [23]. Therefore, there was less chance that the drug's quality and adherence (appropriate use) would have an impact on its effectiveness; nevertheless, other factors may still have an impact.

At baseline, the study participants' immunity was not thoroughly investigated, however, in the current study, seven study subjects (four before day 28 and three after 35 days) had a treatment failure. Of them, 85.7% of participants were younger than 11 years old. The reason for this discrepancy might be, that children's immunity is lower than adults [24]. However, the recurrence of parasites after treatment might be due to anti-malarial drug resistance, relapse, or reinfection [14]. Partial supervision took place for primaquine treatment but not the chloroquine and study participants were not protected from mosquito biting even though, it confirmed (with interview) that, all study participants had bed nets; this is one of the limitations of this study.

The parasite clearance rate in the present study was 94% at day 3, which is consistent with the findings of 95.5% [21], 93.2% [25], and 93.1% [26]. However, the parasite clearance rate of the study participants on day 3 in the present study (94%) was lower than the findings of 98.6% [15], 100% [13], and 100% [8]. Many factors that affect parasite clearance [27], efficacy of the drug [28], and absorbance of the drug [29].

In the present study, 85.7% of the study participants were less than 11 years old, which implies that children may be less immune than adults and that the efficacy may also be affected by the lower efficacy of CQ-PQ treatment. While the probability of absorbance, which affects clearance, is lower in the present study because, before taking the drug, the participants ate biscuits, eating of food before taking the drug increases absorption [11]. In this study, 6% of study participants were positive on day 3, which is less than the study that was done in Myanmar, where 15% were positive on day 3 [30]. In the present study, 93% of study participants had a previous malaria infection. Repeated malarial infection is important for hosts to increase partial immunity [28] which might increase the clearance of the parasite. This difference might also be due to a combination therapy (CQ plus PQ) in the current study, which means there may be a synergistic effect to clear the parasite, but it needs further comparative study.

The gametocyte clearance rate in this study was 100% on day 2, and it was similar to the study that was done in Shiwarobit, where 100% of participants cleared their gametocytes [13]. The clearance rate in the current study was higher than other findings of 96.5% [15], and 88.5% [31], this difference may be due to either the effectiveness of the drug or the difference in baseline gametocyte density. In the present study, the mean gametocyte count was 290.5 on the day of admission, but the density of gametocytes was not clearly stated in other studies [13, 15, 31].

In the current study, the fever clearance rate (Table 5) of the participants was 98% on day 3, which was consistent with the finding of 98% [25], but the participants in this study had a higher fever clearance rate than the findings of 91% [30], 90.7% [13]. Even though these findings, did not indicate the association between parasite clearance and fever clearance, in the present study there was a significant association between fever clearance and parasite clearance (P = 0.033) on day 3. Fever occurs due to the rupture of blood schizonts and the release of merozoites and toxins (glycosylphosphatidylinositol, DNA-associated haemozoin, and cholesterol-triglycerides), which lead merozoites to infect new blood cells, and due to anti-toxin immune responses [32]. This implies that when the parasites are cleared early, the probability of fever decreases. Thus, may be that early parasite clearance in the present study boosts the fever clearance rate. In addition to this, for a similar reason, fever clearance rates in the present study were lower than those in the study that was done in Arbaminch, which cleared 100% of the fever on day 3, since the parasite clearance rate was 100% on day 2 [8].

Based on a paired t-test there was a significant improvement in the mean haemoglobin level between follow-up days of 11.9–12.7 g/dl (between day 0 and day 42, P < 0.001), which was similar to [8] 11.8–13.4 g/dl (P < 0.001) between day 0 and day 42. However, the baseline prevalence of anaemia (56%) of the study participants in the present study was higher than the studies in Shiwarobit (27.9%) [13], Southern, Ethiopia (18.5%) [33]. Malaria-related anaemia occurs due to the destruction of RBCs by parasites and by removing infected RBCs with immune cells [34]. Younger children produce lower amounts of pro-erythropoietin cytokine and low expression of complement regulatory protein on their RBCs, which are important for the inflammatory response [35, 36] since this might lead to increased parasitaemia (or less reduction of parasites by these cytokines and proteins). In the present study, the majority of study participants were younger children (85.7%), which might cause high parasitaemia in the blood. This also causes a high destruction of RBCs by parasites and a higher removal of infected RBCs by immune cells.

In this study, on the first day of enrollment, fever, headache, joint pain, and abdominal pain were the most common clinical signs and symptoms, accounting for 100%, 94%, 40%, and 19%, respectively. After treatment, some adverse events were observed: mouth ulcers (1%), coughs (1%), abdominal pain (2%), and nausea (1%). These adverse events are similar to those mentioned by the WHO [37]. These adverse events were also similar to those reported by Belay et al. [13], but unlikely anorexia, behavioural change, dizziness, and blurred vision were not observed in the current study, and there were also no serious adverse events or hypersensitivity reactions up to the end of the follow-up period.

## Conclusions and recommendations

The findings of this study revealed that, co-administration of chloroquine with primaquine is efficacious and well-tolerated with fast resolution of fever and a high parasite clearance rate. However, further monitoring of the efficacy of chloroquine plus primaquine treatment for vivax malaria by controlling each drug may be required. Direct supervision of each drug may be required for strong recommendations.

## Data Availability

Data can be accessed from the Ethiopian Public Health Institute.
